# The effects of swimming training at different temperatures along with cinnamon supplementation on liver enzymes and thyroid hormones in diabetic rats

**DOI:** 10.22038/AJP.2023.23248

**Published:** 2024

**Authors:** Sepehr Arammi, Massoud Sahragard, Asiye Seyed, Omidreza Salehi, Seyed Ali Hosseini, Zahra Mosallanezhad

**Affiliations:** 1 *Department of Sport Physiology, Behbahan Branch, Islamic Azad University, Behbehan, Iran*; 2 *Department of Physical Education and Sport Sciences, University of Kurdistan, Sanandaj, Iran*; 3 *Department of Sport Physiology, Marvdasht Branch, Islamic Azad University, Marvdasht, Iran*; 4 *Department of Sport Physiology Zand Institute of Higher Education, Shiraz, Iran*

**Keywords:** Cinnamon, Diabetes mellitus, Exercise, Liver function tests, Temperature, Thyroid hormones

## Abstract

**Objective:**

The aim of this study was to investigate the effects of swimming (S) training in water at 5°C (S5C) and 35°C (S35C) along with cinnamon (Cin) supplementationon liver enzymes and thyroid hormones in streptozotocin (STZ(-induced diabetic rats.

**Materials and Methods:**

In this experimental trial, 48 diabetic rats (55 mg/kg STZ) were divided into (1) diabetic control (CD), (2) S5C, (3) S5C+Cin, (4) S35C, (5) S35C+Cin and (6) Cin groups.Eight rats were placed in the healthy control (HC) group to evaluate the effects of diabetes induction on the research variables. Swimming training was performed at 5±2°C and 35±2°C for eight weeks, 3 days a week.For Cin supplementation, 200 mg/kg/day of the aqueous extract of cinnamon was dissolved in the animals drinking water. One-way analysis of variance with Tukey's *post- hoc* test in Graphpad Prism software was used to analyze the findings.

**Results:**

S5C and S35C significantly increased thyroid-stimulating hormone (TSH), and decreased alkaline phosphatase (ALP) and alanine aminotransferase** (**ALT)(p≤0.05). TSH levels in the S35C group were higher than the S5C group (p≥0.05); ALT levels in the S5C group were lower than the S35C group (p≥0.05). Also, Cin decreased AST and ALT levels (p≥0.05), while S35C+Cin decreased T3, ALP and ALT and S5C+Cin decreased ALP (p≥0.05).

**Conclusion:**

It seems that training at different temperatures and consumption of cinnamon synergistically lead to improvement of liver enzymes and modulation of thyroid hormones. However, the effect of training in cold water and its impact on thyroid hormones is still unknown and needs further research.

## Introduction

Diabetes is a common metabolic disorder characterized by impaired glucose, insulin, endocrine, and metabolic profile(Hosseini et al., 2020). Defects in the metabolism of energy substrates in these individuals are associated with the development of various diseases, including cardiovascular disease, and renal, hepatic and thyroid disorders (Davari et al., 2020). Also, lifestyle changes, decreased physical activity and impaired insulin function increase reactive oxygen species and inflammatory factors, reduce the ability of liver cells in mitochondrial biogenesis, and initiate apoptosis in various tissues of the body ( Davari et al., 2020; Hosseini et al., 2020). 

In addition, the rate of thyroid disorders in people with diabetes and metabolic syndrome is 2 to 3 times that of healthy people (Kalra et al., 2021; Xing et al., 2021). With disturbing thyroids hormone levels, diabetes can lead to impaired immune systems and hypothalamic-pituitary-thyroid dysfunction, increased interleukin-6 (IL-6) levels, impaired caloric intake, disruption in activity of thyroxine (T4) and triiodothyronine (T3) hormones (Xing et al., 2021). As a result, T4, T3, Free T3, Free T4, and thyroid stimulating hormone (TSH) levels decrease in patients with diabetic ketoacidosis while reverse T3 (rT3) levels increase (Xing et al., 2021). Given the importance of physical activity in regulating blood glucose and insulin as well as improving the metabolism of energy substrates in diabetes, many researchers have reported the role of regular physical activity in improving mitochondrial biogenesis and cellular redox in the liver tissue as well as improving fat profile, liver enzymes and thyroid hormone function (Davari et al., 2020; Hosseini et al., 2020; Hosseini et al., 2018, 2020; Teixeira et al., 2020; Zar et al., 2016). However, due to the diversification of exercise in different environments and temperatures, exercise in cold and hot temperatures has different effects on metabolism. So that researchers have pointed out that cold exposure can lead to the activation of thyroid hormones, increase in thermogenesis and cold compensation (Lisboa et al., 2003), and hence, exposure to a temperature of 4°C decreased thyroid hormones and increased TSH levels (Lisboa et al., 2003). Exposure to temperatures of 16 to 17°C for ten days increased insulin sensitivity in patients with type 2 diabetes (Remie et al., 2021). In addition, in another study, researchers stated that exposure to cold in elderly rats led to an increase in T3 and T4 compared to the young group. However, there was no significant difference in TSH and TSH receptor values in young and elderly groups. In general, they concluded that exposure to cold temperature leads to improved thyroid hormone function (Park et al., 2017). Also researchers stated that exposure to cold in obese adults and patients with type 2 diabetes can lead to the release of myokines, improved insulin sensitivity, and ultimately increased metabolism of energy substrates (Ivanova and Blondin, 2021). In addition, in a study, the results showed that exposure to a temperature of 16-17°C for 10 days led to an improvement in the metabolism of glucose and lipid in patients with type 2 diabetes (Remie et al., 2021). Despite the favorable results following regular physical activity, there is limited information on the effect of physical activity in cold temperatures compared to hot temperatures.

On the other hand, given the irreversible side effects of synthetic drugs, the use of herbs is recommended to patients with diabetes due to fewer side effects (Ghanbari et al., 2022). Among these medicinal plants, cinnamon with the scientific name of *Cinnamomum verum*is widely cultivated in different parts of Asia, Australia and South America, and due to its antioxidant, anti-inflammatory, and antimicrobial properties as well as its effects onimproving fat metabolism (Gheflati et al., 2022) and regulating sugar and insulin, it has been effective in improving blood pressure (Shekarchizadeh-Esfahani et al., 2021). In a study, consumption of cinnamon at doses less than 1500 mg/day for 12 weeks had beneficial effects on liver enzymes (Shekarchizadeh-Esfahani et al., 2021) and glycemic indices (Ostadpoor, 2020); also, consumption of 400 mg/kg of cinnamon for 25 days reduced serum T3 levels, and optimally regulated the gene expression of thyroid hormones in rats (Gaique et al., 2016). Given the important role of diet along with physical activity, studies were conducted on the simultaneous effects of training and cinnamon consumption and showed that swimming training and cinnamon consumption improved glucose transporter and insulin receptor gene expression in white adipose tissue of diabetic rats (Karimi Fard et al., 2021). Endurance training and cinnamon consumption improved the activity of antioxidant enzymes in diabetic rats (Monazami et al., 2020). Despite numerous studies, no study has examined the effect of exercise training at different temperatures with cinnamon supplementation on liver enzyme and thyroid hormone levels.

Regarding the simultaneous effect of exercise and cinnamon, the results of a study showed that the combination of these two interventions (swimming exercise and cinnamon supplementation) improves the lipolysis pathway of visceral adipose tissue (Mohammadi et al., 2023). It seems that conducting such studies could provide researchers in the field of nutrition and exercise with more information about the simultaneous effect of temperature, physical activity as well as cinnamon supplementation on liver damage and thyroid hormones markers. Although the role of exercise on glucose metabolism is well established, it seems that the effect of activity at different temperatures on thyroid hormones and liver enzymes, as metabolic indicators, is still unknown. Therefore, it seems necessary to conduct fundamental studies that can lead to more information on the effect of activity at different temperatures along with antioxidants on thyroid and liver hormones. Therefore, the present study aimed to investigate the effect of swimming training at different temperatures with cinnamon supplementation on serum levels of liver enzymes and thyroid hormones in diabetic rats.

## Materials and Methods

In this experimental study, 56 Sprague Dawley rats (10 to 12 weeks old and 210±25 g) were prepared from the Animal Breeding and Reproduction Center of Islamic Azad University, Marvdasht Branch and kept in the animal laboratory of this university for 7 days to adapt to the environment. Then, 48 diabetic rats were subjected to intraperitoneal injection of 55 mg/kg streptozotocin (Sigma USA) and four days later, their blood glucose levels were measured by glucometer 2041 (Germany). Rats with blood glucose above 250 mg/dl were identified as diabetic rats (Hosseini et al., 2020). Based on the fasting blood glucose levels, the diabetic rats were divided into (1) diabetic control (DC), (2) swimming training at 5°C (S5C), (3) swimming training at 5°C and cinnamon consumption (S5C + Cin), (4) swimming training at 35°C (S35C), (5) swimming training at 35°C and cinnamon consumption (S35C+Cin), and (6) cinnamon consumption (Cin) groups. It is noteworthy that 8 rats were sacrificed to investigate the effects of diabetes induction on the research variables in the healthy control (HC) group. It is worth mentioning that there were no deaths in the present study.

**Swimming training**

To demonstrate the ability of rats to perform swimming training in a special pool, the rats first swam in water at 5°C. Next, their activities were closely monitored for 2 min and recorded until the rats swam and tried to get rid of that position. This operation was performed for 6 sessions to familiarize the rats with training conditions. Based on Lubkowska et al. (2019), water swimming at 5 ±2 and 35±2°C was performed in the first week for 2 min, 5 days a week, and then 30 sec was added to each training session until the training duration reached 4 min. Then, the rats trained at 5°C for 4 min until the end of the fourth week. The rats performed training in a special swimming tank with dimensions of 100 cm length, 50 cm width and 50 cm depth (Bryczkowska et al., 2017; Lubkowska et al., 2019). 

**Cinnamon supplementation**

In this study, to prepare an aqueous extract of cinnamon, first 100 g of cinnamon prepared from Marvdasht agricultural Jihad was dissolved in 1000 ml of pure water. The solution was then boiled for ten minutes and after cooling, it was passed through paper strainer No. 1 (solution contained 20% aqueous cinnamon extract). Then cinnamon extract was added to the drinking water of rats so that the everyday drinking water of each cage (5 rats per cage) contained 200 mg/kg of cinnamon (Ismail, 2014).

It is worth mentioning that in order to ensure the consumption of the exact amount of cinnamon in the first weeks, the amount of drinking water of all 5 rats was approximately calculated and during the research period, 210 mg was dissolved in their drinking water daily.

**Dissection and sampling**

Forty-eighthours after the last training session and cinnamon supplementation, and following 16 hrof fasting, rats were anesthetized using ketamine (70 mg/kg) and xylazine (25 mg/kg)(Alfasan Co., Netherlands), and after specialists’ ensuring of complete anesthesia, 5 mlof blood was drawn directly from the heart tissue of rats. Later, the rats were euthanized using the spinal cord cutting method and their carcasses were burned in an incinerator at a temperature of 7000 °C (Gilbert Gedeon, 2016). The blood samples were placed in a room for 2 hrto clot and then centrifuged at 3000 rpm in an 8-channel Hitachi for 12 min to separate the serum. To measure the levels of TSH (ELISA kit-Pars Azmoon, with an accuracy of 0.1 µIU/ml), T3 (with an accuracy of 0.05 ng/ml) and T4 (T4 96 ELISA kit-Pars Azmoon, with an accuracy of 0.15 µg/dl), the ELISA kit, made by Ideal Co. in Iran was used; as well as alkaline phosphatase (ALP), aspartate aminotransferase (AST) and alanine transaminase (ALT) (with an accuracy of U/L) were measured by Pars Azmoon kit (made in Iran). 

**Data analysis procedure**

The Shapiro-Wilk test was used to investigate the normal distribution of the data of the present study. Regarding inferential statistics, one-way analysis of variance was performed to examine the differences between the groups and Tukey's *post hoc* test was performed to evaluate the differences between the groups in Graphpad Prism 8.4.3 software. Also, a significance level of p≥0.05 was considered for data analysis.

## Results

**Figure 1:**
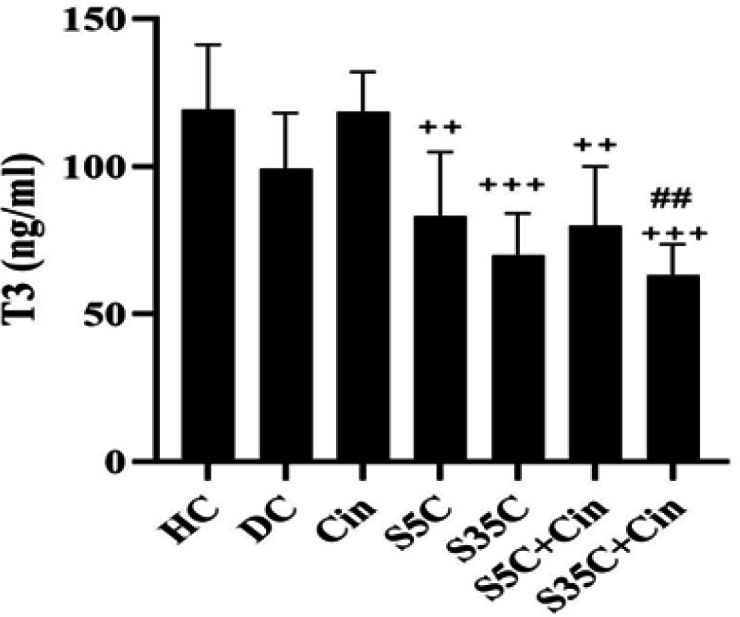
Serum T3 levels in rats in the study groups

HC: Healthy Control; DC: Diabetic Control; Cin: Cinnamomum; S5C: swimming training at 5°C; S35C: Swimming training at 35°C, S5C+Cin: Swimming training at 5°C and cinnamon consumption; S5C+Cin: Swimming training at 5°C; S35C+Cin: Swimming training at 35°C and cinnamon consumption

**Figure 2:**
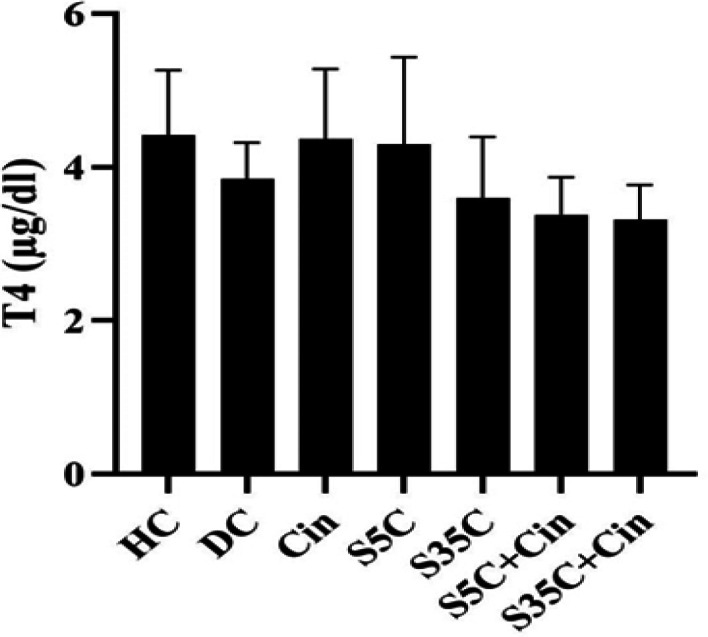
Serum T4 levels in rats in the study groups

HC: Healthy Control; DC: Diabetic Control; Cin: Cinnamomum; S5C: swimming training at 5°C; S35C: Swimming training at 35°C, S5C+Cin: Swimming training at 5°C and cinnamon consumption; S5C+Cin: Swimming training at 5°C; S35C+Cin: Swimming training at 35°C and cinnamon consumption

**Figure 3:**
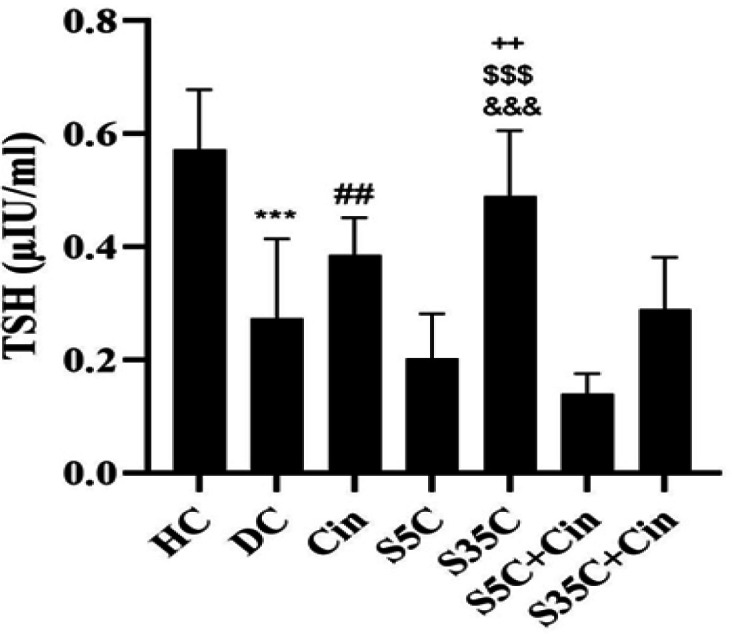
Serum TSH levels in rats in the study groups.

HC: Healthy Control; DC: Diabetic Control; Cin: Cinnamomum; S5C: swimming training at 5°C; S35C: Swimming training at 35°C, S5C+Cin: Swimming training at 5°C and cinnamon consumption; S5C+Cin: Swimming training at 5°C; S35C+Cin: Swimming training at 35°C and cinnamon consumption

**Figure 4:**
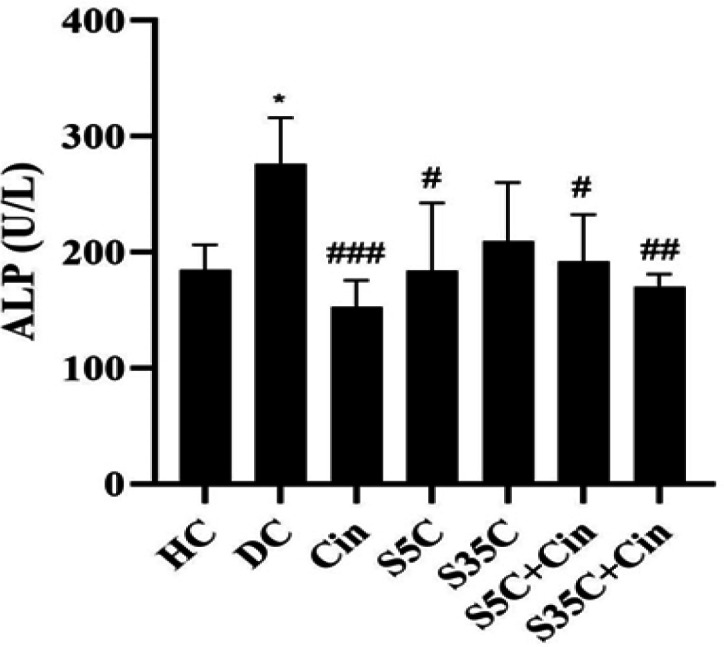
Serum ALP levels in rats in the study groups

HC: Healthy Control; DC: Diabetic Control; Cin: Cinnamomum; S5C: swimming training at 5°C; S35C: Swimming training at 35°C, S5C+Cin: Swimming training at 5°C and cinnamon consumption; S5C+Cin: Swimming training at 5°C; S35C+Cin: Swimming training at 35°C and cinnamon consumption

**Figure 5:**
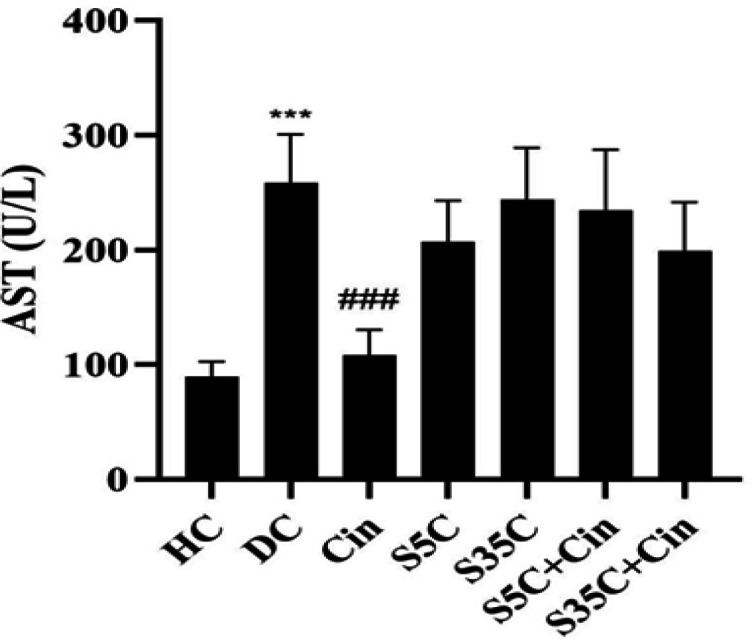
Serum AST levels in rats in the study groups

HC: Healthy Control; DC: Diabetic Control; Cin: Cinnamomum; S5C: swimming training at 5°C; S35C: Swimming training at 35°C, S5C+Cin: Swimming training at 5°C and cinnamon consumption; S5C+Cin: Swimming training at 5°C; S35C+Cin: Swimming training at 35°C and cinnamon consumption

**Figure 6:**
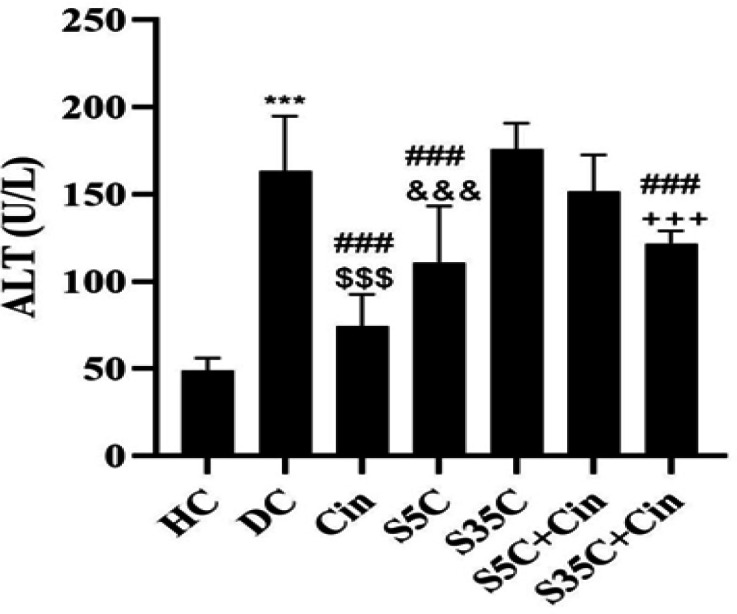
Serum ALT levels in rats in the study groups.

HC: Healthy Control; DC: Diabetic Control; Cin: Cinnamomum; S5C: swimming training at 5°C; S35C: Swimming training at 35°C, S5C+Cin: Swimming training at 5°C and cinnamon consumption; S5C+Cin: Swimming training at 5°C; S35C+Cin: Swimming training at 35°C and cinnamon consumption

The levels of T3, T4, TSH, ALP, AST and ALT are shown in Figure 1-6, respectively. The results of one-way analysis of variance showed significant differences in T3 (p=0.001), TSH (p=0.001), ALP (p=0.001), AST (p=0.001) and ALT (p=0.001) levels among the groups, but no significant difference in T4 levels was observed among the groups (Figure 2).

##p=0.01 shows a significant decrease compared to the DC group++. p=0.01 and+++p=0.001 showa significant decrease compared to the Cin group

The results of Tukey’s *post hoc* test showed that T3 levels in the HC and DC groups were not significantly different (p=0.41), however in the S35C+Cin group, the levels were significantly lower than the DC group (p=0.01); also in the Cin group, the levels were significantly higher than the S5C (p=0.01), S35C (p=0.001), S5C+Cin (p=0.006) and S35C+Cin (p=0.001) groups (Figure 1).

TSH levels in the DC group were significantly lower than the HC group (p=0.001), however in the S35C group these levels were significantly higher than the DC group (p=0.006); TSH levels in the Cin group were significantly higher than the S5C (p=0.02) and S5C+Cin (p=0.001) groups. Also, in the S35C group, the levels were significantly higher than the S5C (p=0.001), S5C+Cin (p=0.001) and S35C+Cin (p=0.014) groups (Figure 3).

***p=0.001. Significant decrease compared to the HC group. ## p=0.05. Significant increase compared to the S5C and S35C+Cin groups. $$$p=0.001. Significant increase compared to the S5C and S5C+Cin groups. &&&p=0.001. Significant increase compared to the S35C+Cin group.

ALP levels in the DC group were significantly higher than the HC group (p=0.02), however, in the Cin (p=0.001), S5C (p=0.013), S5C+Cin (p=0.04) and S35C+Cin (p=0.003) groups, the levels were significantly lowerthan the DC group (Figure 4).

*p=0.05. Significant increase compared to the HC group. #p=0.05. Significant decrease compared to the DC group.

AST levels in the DC group were significantly higher than the CH group (p=0.001). However, in the Cin group, the levels were significantly lower than the DC (p=0.001), S5C (p=0.001), S35C (p=0.001), S5C+Cin (p=0.001) and S35C+Cin (p=0.002) groups (Figure 5).

ALT levels in the CD group were significantly higher than the HC group (p=0.001). However, in the Cin (p=0.001), S5C (p=0.002) and S35C+Cin (p=0.02) groups, the levels were significantly lower than the DC group. Also, in the Cin group, the levels were significantly lower than the S35C (p=0.001), S5C+Cin (p=0.001) and S35C+Cin (p=0.005) groups. ALT levels in the S5C group were significantly lower than the S35C (p=0.001) and S5C+Cin (p=0.03) groups, and in the S35C+Cin group were significantly lower than the S35C group (p=0.001) (Figure 6).

***p=0.001. Significant increase compared to the HC group. ###p=0.001. Significant decrease compared to the DC, S5C, S35C, S5C+Cin and S35C+Cin groups.

***p=0.001. Significant increase compared to the HC group. ###p=0.001. Significant decrease compared to the DC group. $$$p=0.001. Significant decrease compared to the S35C, S5C + Cin and S35C + Cin groups. &&&p=0.001. Significant decrease compared to the S35C and S5C + Cin groups. +++p=0.001. Significant decrease compared to the S35C group.

## Discussion

The results of present study showed thatin the DC group, TSH levels were significantly lower and ALP, AST and ALT values were significantly higher than the HC group and TSH levels in the S35C group were significantly higher than DC and S5C groups. Studies show that hypothyroidism is a common disorder in diabetic patients but it seems that after the disturbance of the metabolism of fats (Hage et al., 2011), hypothyroidism is associated with a disturbance in the immune system, which finally, with the attack of the anti-thyroid peroxidase (TPO) antibody to the tissue of the thyroid gland, causes a disturbance in the transcription of TSH and a decrease in the expression of T3 and T4 (Veisi et al., 2018). However, conflicting results have been reported regarding TSH changes, which have reported both an increase (Veisi et al., 2018) and a decrease (Eom et al., 2022) in diabetic patients. In a study, researchers pointed out that circulating levels of integrins can affect TSH levels so that the glucagon-like peptide-1 receptor (GLP-1R) agonist, exenatide, and neurotoxicity caused by neurotoxins such as streptozotocin (STZ) in the long term (for example, more than 6 months) can also reduce TSH levels (Eom et al., 2022). Exercise is considered a challenge to disrupt the body's homeostasis, which can lead to damage to various organs; however, performing intensity-dependent exercise with the mechanism of increasing levels of catecholamines and androgens changes the function of the hypothalamic-pituitary-thyroid (HPT) axis, metabolic hormones secreted by the endocrine glands and other tissues (Teixeira et al., 2017). These hormonal changes increase thyrotropin (TSH) levels, and this hormone activates the thyroid gland by trapping iodine, causing T4 synthesis; T4 is then diodinized and converted to T3 and, if necessary, enters the bloodstream and affects various cells (Fortunato et al., 2008; Teixeira et al., 2017). 

Various results have been reported in relation to the effect of exercise on thyroid hormones. In a study, T3 levels increased immediately after 20 min of exercise with 75% of maximal oxygen consumption, but no change in T4 and TSH levels was observed (Fortunato et al., 2008). Also, following a session of acute exercise activity with 60% of intensity, T3 levels were associated with an increase in androgenic levels, while in a higher intensity activity, T3 levels were associated with an increase in catecholamines (Coggan et al., 2000). Adaptation to exercise activities after long-term training can modulate HPT function by modulating the androgenic hormones as well as catecholamines and increase TSH, augment storage, and improve the function of T3 and T4 hormones (Fortunato et al., 2008; Teixeira et al., 2017). It seems that cold activity or exposure can activate the beta-adrenergic pathway, and the cAMP cascade by stimulating the sympathetic system, increasing thermogenesis, and activating the HPT pathway. Also, in the thyroid gland, it can increase iodine uptake, parathyroid hormone and TSH hormone, and decrease free serum T3 and T4 levels (Kovaničová et al., 2020). 

In a study of cold-acclimatized individuals with winter swimming, 15 min of swimming in ice water increased parathyroid hormone and TSH levels and decreased free T3 and T4 levels (Kovaničová et al., 2020). Also, modulation of thyroid hormones following cold exposure is associated with increased mitochondrial biogenesis and decreased oxidative stress in the liver tissue; (Lubkowska et al., 2019). No comprehensive data is available regarding the effect of cold exposure on liver enzymes, but cold exposure to 14-16°C for 2 hr decreased circulating glutathione activity and increased GSSG in the liver tissue and blood of elderly animals (Teramato et al., 1998). Cold exposure also reduced glutathione peroxidase in the heart and liver tissues and significantly reduced hydrogen peroxide levels in rodents (Augustyniak and Skrzydlewska, 2004). However, exposure to -60 to -90°C increased hepatic catalase and superoxide dismutase levels (Skrzep-Poloczek et al., 2017); also, eight weeks of cold exposure and water swimming for 4 min at 5°C and 36°C increased the antioxidant activity in the serum levels of female rats. Yet comparing swimming temperatures, it was shown that 5°C had a more favorable effect on increasing superoxide dismutase than 36°C (Lubkowska et al., 2019). The results of these studies seem to indicate that exposure to temperature above 10°C have contradictory effects on antioxidant enzymes, while temperatures below 5°C lead to improved antioxidant activity and improved hepatic metabolism. Despite these results, limited information on the effects of cold on liver enzymes suggests further studies in future.

The results of the present study showed that Cin reduced AST and ALT levels in diabetic rats. The mechanism of cinnamon extract on liver enzymes is not yet fully understood, but cinnamon supplementation with the mechanism of activation of the Peroxisome proliferator- activated receptor gamma (PPARγ) can lead to weight improvement and modulation, reducing the activity of inflammatory and proinflammatory factors such as tumour necrosis factor α (TNF-α), interlukin-6 (IL-6), and C-reactive protein (CRP), reduction of reactive oxygen species, increase of liver proteins, increase of insulin sensitivity in white adipose tissue, increase of glucose-4 transporter in adipose tissue, increase of insulin secretion, increase of mitochondrial oxidation of free fatty acids and ultimately improvement of liver enzymes and especially reducing AST levels (Shekarchizadeh-Esfahani et al., 2021). In this regard, in a meta-analysis study, researchers showed that cinnamon extract improves AST, ALP and ALT levels, and the desired effect of cinnamon extract is related to doses above 1500 mg/kg with a duration of less than 8 weeks (Shekarchizadeh-Esfahani et al., 2021). Also, cinnamon consumption, by regulating gene expression in thyroid cells, modulates gene expression in inflammatory cells, increases the gene expression of Phospholamban, decreases the gene expression of SERCA2a and RyR2, and improve the T3 expression signaling pathway (Gaique et al., 2016). Some studies have been performed in this regard, for instance, consumption of cinnamon for 25 days and 400 mg / kg reduced T3 levels and improved the signaling pathway associated with the modulation of thyroid hormones (Gaique et al., 2016). Consumption of eight grams of cinnamon per day for eight weeks significantly reduced the nuclear transcription factor kappa B (NF-kB) and hs-CRP in patients with type 2 diabetes, but no significant effect was observed on TNF-α and IL-6 levels (Davari et al., 2020). Consumption of cinnamon powder at doses of 2 and 4 g/kg/day for 30 days caused weight loss, decreased food intake, triglycerides, LDL-C, cholesterol and increased HDL-C levels in albino rats (Alsoodeeri et al., 2020). Although there are limitations regarding the effect of cinnamon on liver enzymes in diabetes, the results of studies show a favorable metabolic effect of cinnamon on the liver and thyroid tissues.

The present results also showed that S35C + Cin decreased T3, ALP and ALT levels and S5C + Cin decreased ALP levels in rats with diabetes. In addition, increased levels of T3 and TSH, and decreased levels of ALP, AST and ALT were more dependent on Cin consumption and Cin had a more favorable effect than training. Unfortunately no study was found to deal with the simultaneous effect of swimming training at different temperatures and cinnamon consumption on liver enzymes. It seems that swimming training and cold exposure, depending on intensity, training duration and training frequency, with the mechanism of modulation of catecholamines, androgenics, sympathetic system stimulation, increase of thermogenesis, with induction of HPT axis, improvement of antioxidants, and increased levels of gene expression lead to increased parathyroid hormone and TSH, decreased T3 and T4, and improved liver enzymes (Augustyniak and Skrzydlewska, 2004; Fortunato et al., 2008; Kovaničová et al., 2020; R. B. Teixeira et al., 2017; Teramoto et al., 1998). Cinnamon consumption by activating PPARγ, improving weight, reducing the activity of inflammatory and proinflammatory factors such as TNF-α, IL-6, and CRP, reducing reactive oxygen species, regulating gene expression in thyroid cells, improve glucose and fat metabolism and improves the liver enzymes (Shekarchizadeh-Esfahani et al., 2021) and the thyroid gland hormones (Gaique et al., 2016).

Therefore, swimming and consuming cinnamon synergistically improve liver enzymes and thyroid hormones. In this regard, eight weeks of swimming training and cinnamon consumption synergistically improves glucose-4 transporter and insulin receptor in the white adipose tissue of rats with diabetes (Karimi Fard et al., 2021). Cinnamon consumption, aerobic training and concurrent training had the same effect on glycemic indices in patients with type 2 diabetes (Arabmomeni and Haji Hidari, 2019); also, exercise combined with cinnamon consumption improved cholesterol and some lipid indicators in rats with type 1 diabetes (Moura et al., 2010). Previous studies have shown that other herbs such as crocin along with exercise, by increasing mitochondrial biogenesis pathway and modulating liver cell redox, improve liver function in rats with type 2 diabetes (Davari et al., 2020). Also, considering the modulating effect of cinnamon after swimming training at different temperatures, exercise training can be recognized as a metabolic challenge, so that various studies of liver and metabolic enzymes following exercise have obtained different results (Kovaničová et al., 2020). Given the presence of baseline metabolic disorders in diabetic patients, cold exposure seems to lead to thyroid hormone disorders in them, as a study showed that exposure to cold water for 15 min by disrupting calcium levels led to decreased parathyroid hormone and TSH levels (Kovaničová et al., 2020); however, given long-term acclamation to cold exposure and activation of the catecholamine and cAMP pathways, it appears that cold-related metabolism of fats and liver enzymes becomes more affected (Kovaničová et al., 2020). In justifying the more favorable effect of cinnamon than exercise on variables, studies have shown that oxidative stress caused by moderate and long-term exercise is a factor in creating metabolic adaptations through the regulation of leptin, ghrelin, and adiponectin hormones, improving the function of the hypothalamus and the pituitary gland; However, high levels of oxidative stress can increase these disorders and disrupt the regulation of the expression of some metabolic genes in brain tissue (Magherini et al., 2019). On the other hand, it seems that cinnamon supplement as an antioxidant can moderate the effects of exercise on oxidative stress. Therefore, it seems that in this study, the improvement of some metabolic enzymes following the consumption of cinnamon compared to exercise is attributed to the stronger antioxidant effects of cinnamon. Therefore, the superior effect of hot water training over cold water on AST levels can depend on this postulation. Considering the role of apolipoproteins and fat profile on changes in liver enzymes, it seems that not evaluating these variables is one of the limitations of the present study. Thus, in the following studies, the role of these variables following training in different temperatures should be evaluated. Also, considering the role of parathyroid hormone (parathormone), iodine and metabolic axes of the nervous system, it seems that the lack of study of these pathways is another limitation of the present study. Evaluate both the liver and the thyroid gland. Thus, it is suggested that in future studies, the levels of parathyroid hormone, SERCA2a, RyR2, PPARγ pathway in both liver and thyroid gland be evaluated. In addition, it seems that due to the effectiveness of catecholamines and myokines following exercise and their effect on the nervous system and endocrine secretion, the lack of evaluation of these variables is one of the limitations of the present study. It also seems that the lack of evaluation of TSH activating hormone in the hypothalamus tissue to understand the path of the nervous system of these changes is one of the other limitations, and it is suggested to evaluate these variables in future studies. It seems that training at different temperatures and consumption of cinnamon synergistically lead to improvement of liver enzymes and modulation of thyroid hormones. Also, the effect of training in cold water and its impact on thyroid hormones is still unknown and needs further research.

## Conflicts of interest

The authors have declared that there is no conflict of interest.
